# Supplementary material for: Genotype-by-environment interactions shape ubiquitin-proteasome system activity
Source: Genetics. 2025 Aug 29;231(3):iyaf180. doi: 10.1093/genetics/iyaf180 (PMC12606439; doi:10.1093/genetics/iyaf180)
Supplement: iyaf180_Supplementary_Data [file iyaf180_supplementary_data.zip › Supplementary_Figures.pdf]

Supplementary Figures for

# Genotype-by-environment interactions shape ubiquitin-proteasome system activity

Randi R. Avery, Mahlon A. Collins, Frank W. Albert

Department of Genetics, Cell Biology, & Development

University of Minnesota

Minneapolis, MN 55455

USA

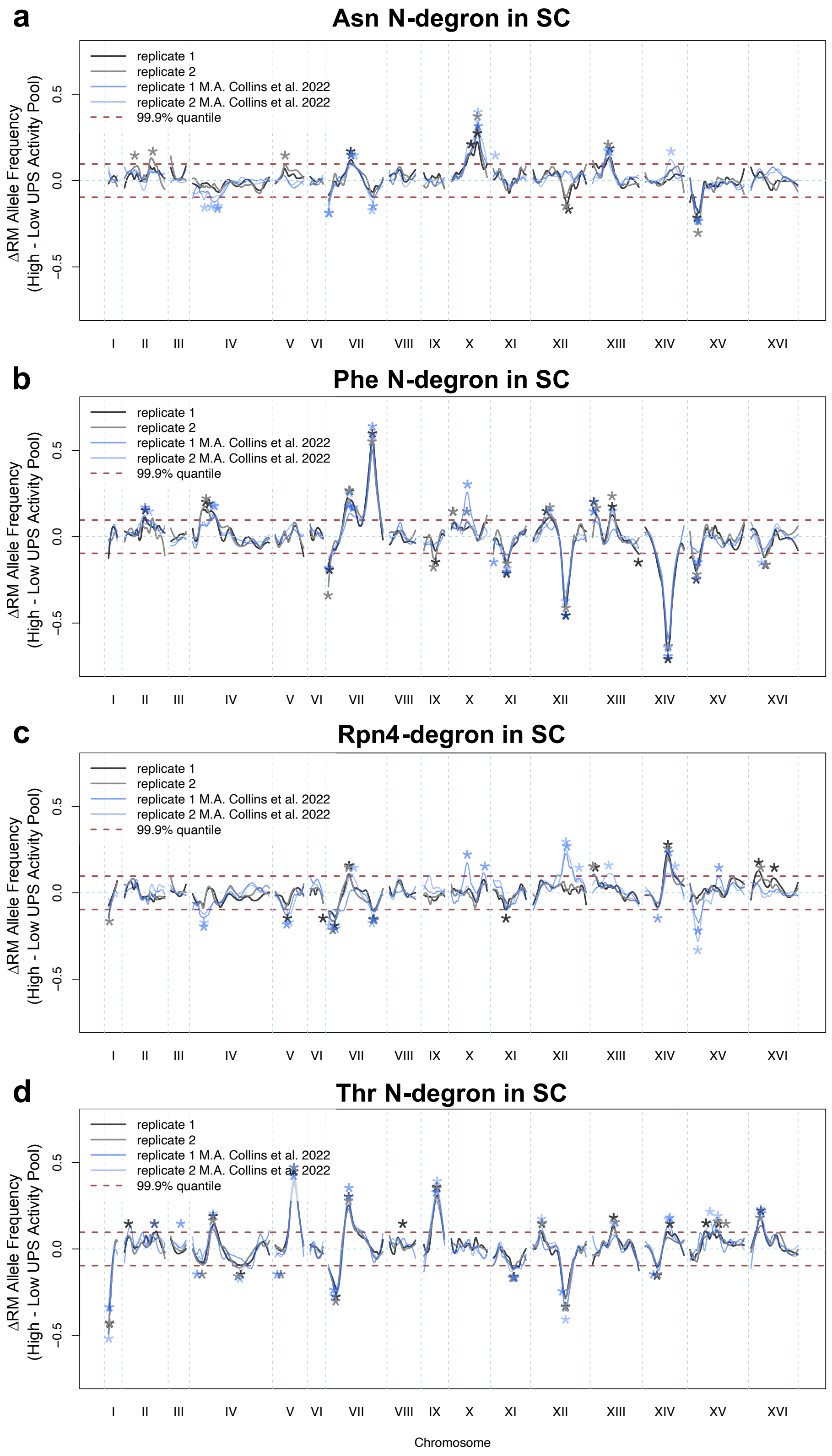


**e**

| Reporter | Total QTLs present in both studies | Total QTLs in current study | Percent Replicated |
|---|---|---|---|
| Asn | **4** | 5 | **80** |
| Phe | **11** | 14 | **79** |
| Rpn4 | **3** | 6 | **50** |
| Thr | **12** | 14 | **86** |

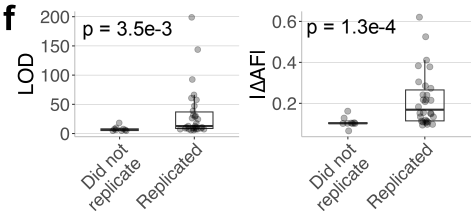

**Supplementary Figure 1: QTL reproducibility. a-d)** QTL traces for the four reporters measured in SC in this study and (Collins et al. 2022, 2023). **e)** Table summarizing the number of QTLs that replicated between studies. **f)** QTLs that did not replicate between studies (n = 9) had significantly lower LOD scores and absolute allele frequency differences than QTLs that did replicate (n = 30). T-test p-values are shown.

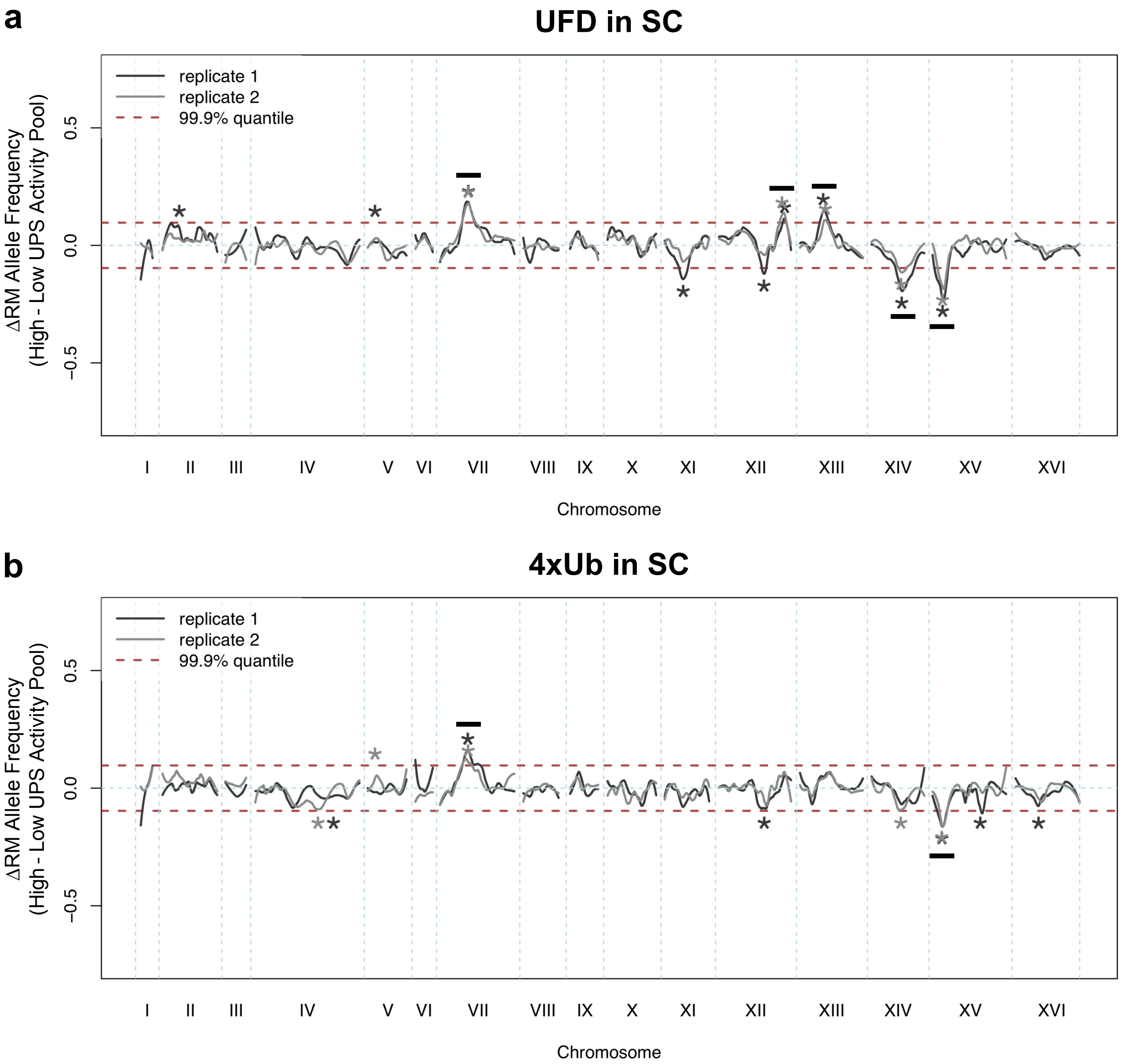


**Supplementary Figure 2: QTLs for 4xUb and UFD in SC**. The plots show the loess-smoothed allele frequency difference between the high and low UPS activity pools across the genome for each of two independent biological replicates. Asterisks denote QTLs, defined by allele frequency differences that exceed an empirically-derived LOD score significance threshold in the given replicate. Horizontal black lines indicate QTLs that were present in both replicates. The dashed red horizontal lines denote an empirically-derived 99.9% quantile of the allele frequency difference. **a)** UFD reporter in SC. Five QTLs were present in both replicates. **b)** 4xUb reporter in SC. Two QTLs were present in both replicates.

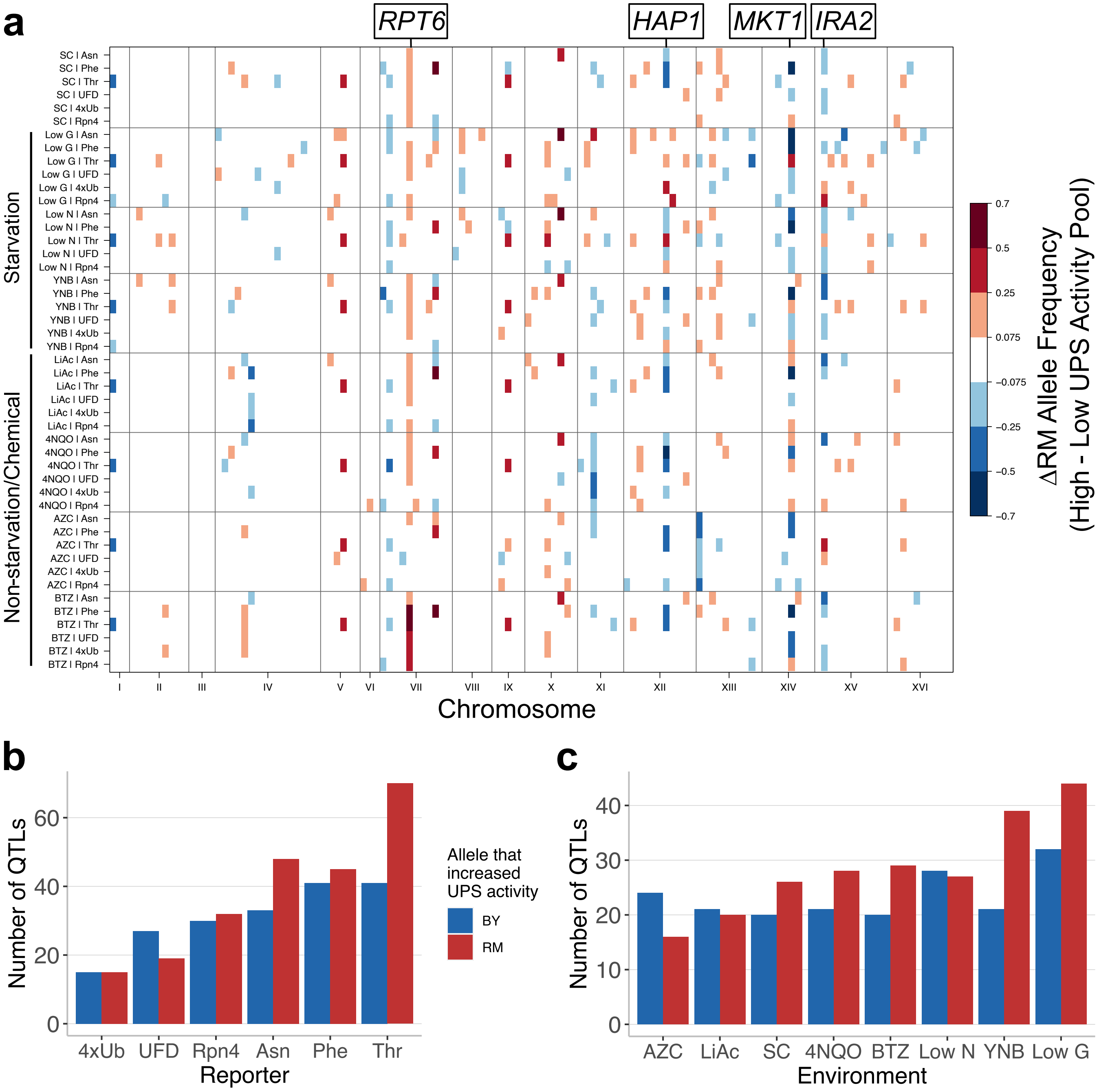


**Supplementary Figure 3: UPS activity QTLs across environments and reporters. a)** QTLs for each reporter / environment combination. Data as in Fig. 3a, but reorganized by environment. Colored blocks denote genomic bins that contain QTLs detected in each of two independent biological replicates, colored according to the direction and magnitude of the effect size, expressed as the RM allele frequency difference between high and low UPS activity pools. Genes in regions discussed in the text are indicated. **b)** Barplot showing the number of times the BY or RM allele increased degradation in the 416 QTLs, by reporter. **c)** Data as in b, but reorganized by environment.

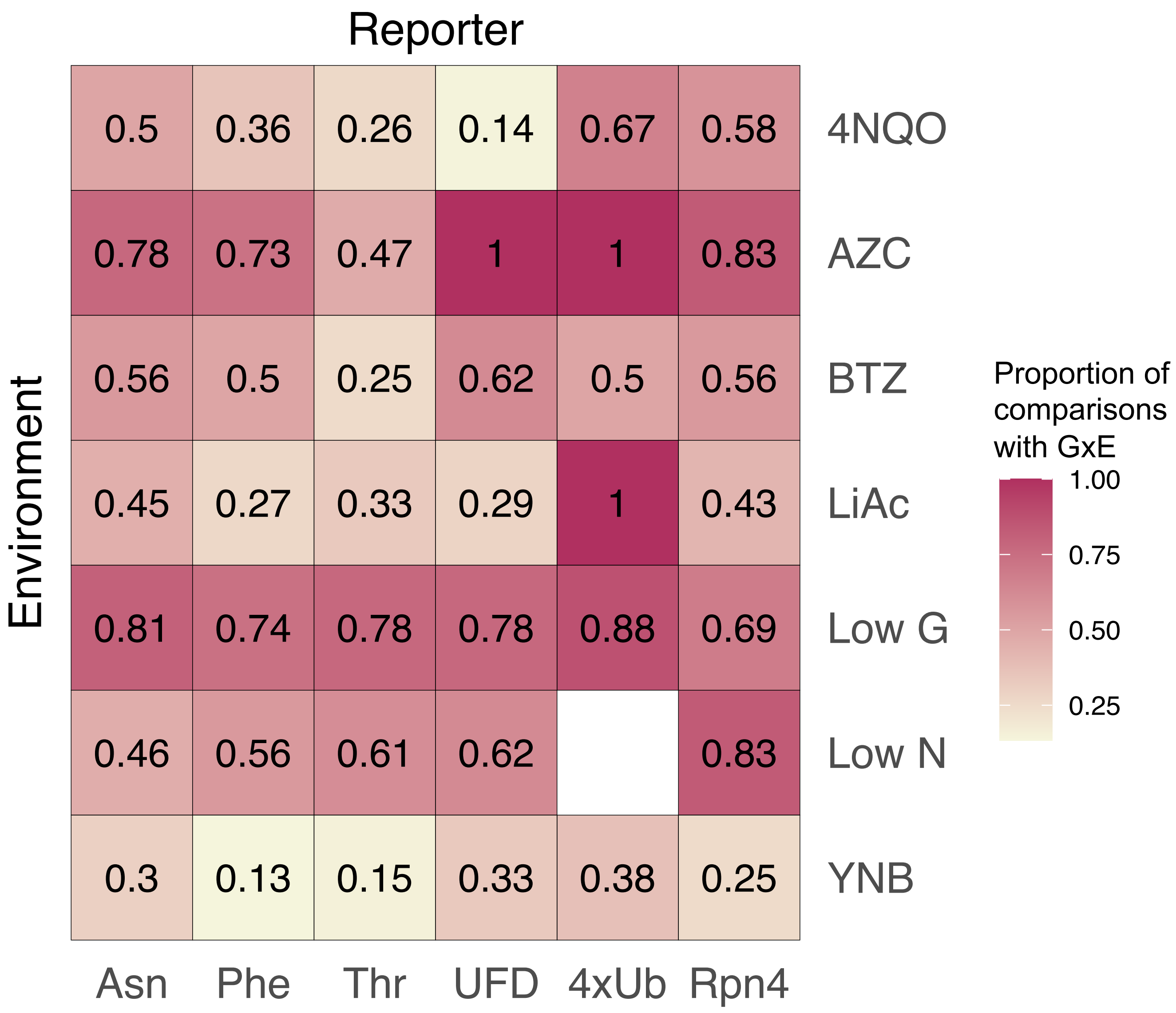


**Supplementary Figure 4:** A heatmap showing the proportion of QTL comparisons that showed GxE out of all comparisons, for combinations of reporters and environments. The UPS activity of the 4xUb reporter could not be reliably measured in low nitrogen, so no data was collected (see Methods).

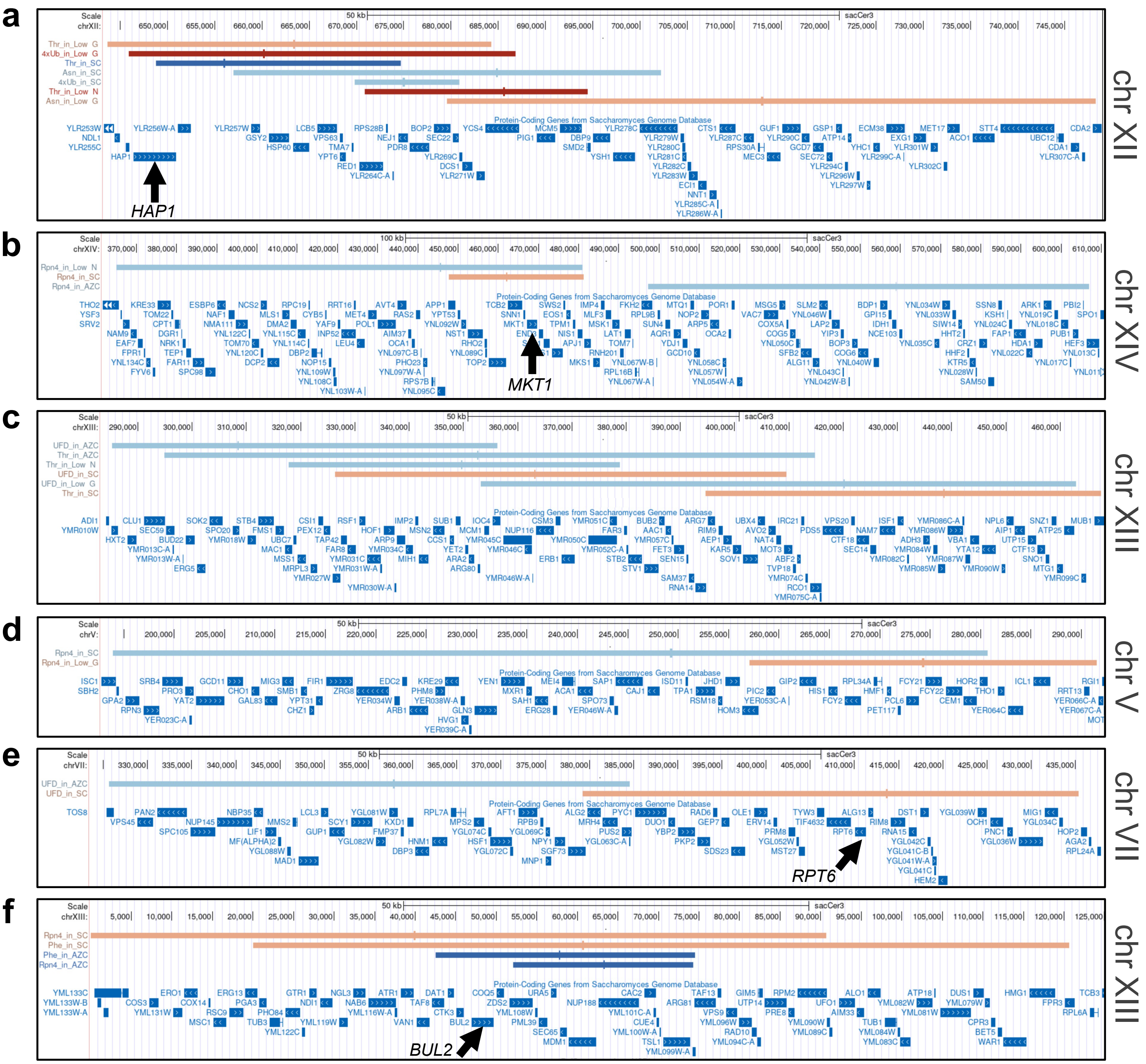


**Supplementary Figure 5: Locations of QTLs within sign change pairs.** Locus plots of the 17 cases of sign change GxE (along with Fig. 5c). The confidence interval (horizontal bars) and peak position (short vertical lines) of the 29 unique QTLs involved in sign changes are shown along the *S. cerevisiae* genome (sacCer3) using UCSC Genome Browser (Nassar et al. 2023). For the QTLs, colors indicate direction and strength of effect as in Fig. 3a. Genes discussed in the text are denoted with black arrows.
